# The bacteriophage WORiC is the active phage element in *w*Ri of *Drosophila simulans *and represents a conserved class of WO phages

**DOI:** 10.1186/1471-2180-11-251

**Published:** 2011-11-15

**Authors:** Jennifer A Biliske, Philip D Batista, Chantalle L Grant, Harriet L Harris

**Affiliations:** 1 Department of Biological Sciences University of Alberta CW 403 Biological Sciences Building Edmonton, Alberta T6G 2E9, Canada; 2 Department of Biology and Environmental Science Concordia University College of Alberta 7128 Ada Boulevard Edmonton, Alberta T5B 4E4, Canada

## Abstract

**Background:**

The alphaproteobacterium *Wolbachia pipientis*, the most common endosymbiont in eukaryotes, is found predominantly in insects including many *Drosophila *species. Although *Wolbachia *is primarily vertically transmitted, analysis of its genome provides evidence for frequent horizontal transfer, extensive recombination and numerous mobile genetic elements. The genome sequence of *Wolbachia *in *Drosophila simulans *Riverside (*w*Ri) is available along with the integrated bacteriophages, enabling a detailed examination of phage genes and the role of these genes in the biology of *Wolbachia *and its host organisms. *Wolbachia *is widely known for its ability to modify the reproductive patterns of insects. One particular modification, cytoplasmic incompatibility, has previously been shown to be dependent on *Wolbachia *density and inversely related to the titer of lytic phage. The *w*Ri genome has four phage regions, two WORiBs, one WORiA and one WORiC.

**Results:**

In this study specific primers were designed to distinguish between these four prophage types in *w*Ri, and quantitative PCR was used to measure the titer of bacteriophages in testes, ovaries, embryos and adult flies. In all tissues tested, WORiA and WORiB were not found to be present in excess of their integrated prophages; WORiC, however, was found to be present extrachromosomally. WORiC is undergoing extrachromosomal replication in *w*Ri. The density of phage particles was found to be consistent in individual larvae in a laboratory population. The WORiC genome is organized in conserved blocks of genes and aligns most closely with other known lytic WO phages, WOVitA and WOCauB.

**Conclusions:**

The results presented here suggest that WORiC is the lytic form of WO in *D. simulans*, is undergoing extrachromosomal replication in *w*Ri, and belongs to a conserved family of phages in *Wolbachia*.

## Background

*Wolbachia pipientis *is an obligate bacterial endosymbiont of insects with a wide distribution. It is a member of the order Rickettsiales and is closely related to the insect vectored mammalian pathogens *Anaplasma *and *Ehrlichia*. Ten supergroups of *Wolbachia *have been identified within the species *W. pipientis *[1]. Supergroups A and B are common insect symbionts which probably diverged from one another 50-60 MYA [2]. The rapid spread of *Wolbachia *through insect populations is enhanced by symbiont-driven modifications to normal host reproductive patterns which are manifested as cytoplasmic incompatibility (CI), parthenogenesis, male killing and feminization (reviewed in [3]).

The possibility of genetically transforming fastidious obligate intracellular bacteria and targeting them to insect vectors of human disease has stimulated renewed interest in *Wolbachia*'s bacteriophage WO. The *Wolbachia *of *Drosophila simulans*, *w*Ri, has acquired four prophage elements that are integrated into the bacterial genome as 18- to 77-kb sequences, termed *w*Ri-WO-A, *w*Ri-WO-B (two identical copies) and *w*Ri-WO-C [4]. In contrast *w*Mel, found in *Drosophila melanogaster*, has one WO-A, one WO-B and a small pyocin-like element. All of these prophage elements are integrated into the *Wolbachia c*hromosome at unique sites. Masui *et al *[5] were the first to demonstrate the existence of the prophage WO in *Wolbachia *of the cricket *Teleogryllus taiwanemma *and later in *D. simulans *(*w*Cof, *w*Ri), the moths *Ephestia kuehniella *(*w*CauB, *w*CauA, *w*Kue, *w*Sca) and *Corcyra cepharonica *(*w*Cep) [6] by electron microscopy and PCR. The WO prophages from *Wolbachia *infecting *D. simulans, D. melanogaster, Culex pipiens, T. taiwanemma, Nasonia vitripennis *and *E. kuehniella *have been sequenced [4,6-12]. WO phage genome sequences from *w*Ri, *w*Mel, and *w*Pip are inferred from their respective bacterial chromosome genome sequencing projects. WOcauB2 and WOcauB3 are two strains of WO phages infecting *Wolbachia *of *E. kuehniella *that have been sequenced from the lytic phase [9]. WOcauB2 has a genome of 43,016 bp encoding 47 predicted open reading frames (ORFs), whereas WOcauB3 has a genome of 45,078 bp and 46 predicted ORFs. With respect to WO phages, little is known about their gene expression, lytic activity, or influence on the phenotypic properties of their hosts.

The nomenclature surrounding the WO phages from different *Wolbachia *strains varies. Originally, the phage found in *w*Kue was tentatively named WO [5], irrespective of how many types of integrated prophages were present. When *w*Mel was sequenced [10], the two prophage inserts were named WO-A and WO-B respective to the origin of replication. Two phage types in *w*Ri, WO-A and WO-B, were named based on sequence homology to the wMel phages, with the addition of one more phage type, WO-C [4]. WOPip is present as five integrated copies in the *Wolbachia *of *C. pipiens *and these are designated WOPip1 through 5 [7]. They have been reported to be more closely related to WO-B of *w*Mel than WO-A of *w*Mel [7].

Bacteriophages are believed to be the mobile genetic elements responsible for the high level of genetic diversity in *Wolbachia *[[10,13] and [14]] through lateral transfer between co-infecting strains. As in other prokaryotes, prophage integration and transformation in *Wolbachia *appear to be major sources of lateral gene acquisition [15]. A group of genes present in the *w*Ri and *w*Mel prophage WO-B genome is most similar to genes found in *Rickettsia *[14], suggesting that interspecies horizontal transfer mediated by phages has also occurred in an insect harboring both bacteria.

Bacteriophages can influence the level of virulence of bacterial pathogens [16] and can change the phenotypic properties of closely related strains of bacteria. In *Wolbachia*-infected *Drosophila*, *Culex, Nasonia *and other insects, WO prophages appear to be temperate, that is, they have an integrated prophage form and can also generate virions which result in bacterial lysis [6,11,15,17] and [18]. In the parasitoid wasp, *N. vitripennis*, Bordenstein *et al *used a quantitative PCR assay to demonstrate that *Wolbachia *titer, which correlates with CI intensity, is inversely related to copy number of temperate WOVitA [15]. This relationship, known as the Phage Density Model, predicts that low CI strains of *Wolbachia *will have a high number of phage particles, and, conversely, high CI strains of *Wolbachia *will have low titers of phage particles [15,19]. In *Drosophila*, however, it is not known which of the diverse prophage elements give rise to lytic viruses, how their lytic properties are regulated, or the effect of lysis on host phenotype. Although most tailed bacteriophages have evolved a temperate lifestyle, it is not yet known if the prophage elements in *w*Ri are functional, defective, satellite phages, or agents of gene transfer [20]. Typically, mature WO phage particles are detected using primers specific to the open reading frame encoding a putative minor capsid protein C (ORF7) [5]. In *w*Ri of *D. simulans*, however, ORF7 is present in all four prophage insertions [WRi_005560], [WRi_007170], [WRi_010220], and [WRi_012630] and so the presence of ORF7 is not a specific indicator of which phage is active.

In this paper we measure the relative copy number of mature, active WORiC phage particles in whole flies and tissues of *D. simulans *and determine variations in *Wolbachia *and WO copy number between individual larval hosts by quantitative PCR. A comparison of the genome architecture of known active phages WOVitA1 and WOCauB2 to WORiC identifies modules for head assembly and DNA packaging as well as tail morphogenesis that are conserved in all known active WO phages.

## Methods

### Strains and media

*D. simulans *(Riverside) (DSR) stocks were maintained at room temperature on a standard diet of cornmeal, dextrose and yeast. Stocks were stably infected with a single *Wolbachia *strain (*w*Ri) and have been maintained at the University of Alberta laboratory for approximately 6 years. The presence of *Wolbachia *was confirmed at regular intervals using 81F and 691R *wsp *primer pairs [21].

### DNA extractions

DNA from whole flies and gonads was extracted from animals that were less than 5 days post eclosion. Newly eclosed flies were separated by sex and allowed to develop to the appropriate age. Gonad DNA samples were obtained from 4 groups of 100-150 testes each and 4 groups of 75-150 ovaries each. Whole fly DNA was taken from 4 groups of 15 flies each. Three groups of 15-minute AEL (after-egg-laying) embryos were obtained by allowing females to oviposit on egg laying dishes made from fruit juice. Embryos were collected and chilled every 15 minutes until approximately 200 μl of packed embryos were obtained per replicate. The eggs were stored at -80C until DNA extractions could be performed.

Synchronization of larvae was accomplished by allowing several hundred females to oviposit on egg-laying dishes for one hour. The eggs were collected and seeded onto standard media. From these, third instar (3') larvae were collected and stored at -80C until DNA extraction.

DNA was extracted from all tissues and flies with the DNeasy Blood and Tissue Kit (Qiagen) using the manufacturer's protocol with an extended, overnight proteinase K digestion. DNA purity and concentration was determined using a Nanodrop ND1000.

### Quantitative PCR for relative copy number

Relative copy numbers of *Wolbachia *and WO phage in *.D. simulans *were obtained using the MiniOpticon System (Bio-Rad). The relative *Wolbachia *infection level was measured by comparing the copy number of the gene for *Wolbachia *surface protein, *wsp*, to a single copy gene in the *Drosophila *genome, CuZn superoxide dismutase (*sod*). Phage copy numbers were measured by comparing the adenine methyltransferase (*wMTase*) (WORiB), *lyzozyme *(WORiA), and *tail tube protein *(WORiC) genes to *wsp *in *w*Ri (see table 1 for locus tags and primer sequences).

**Table 1:** Primer sequences used in this study

ORF Product	Locus Tag	Specificity	Sequence (5'-3')
**Superoxide**	**Dsim GD12822**	***D. simulans***	**F - GTCGACGAGAATCGTCACCT**
**Dismutase (SOD)**			**R - GGAGTCGGTGATGTTGACCT**

**Surface Antigen**	**WRi 010990**	***Wolbachia***	**F - ATCAGGGTTGATGTTGAAGG**
**Wsp (Wsp)**		***w*Ri**	**R - CAGTATCTGGGTTAAATGCTG**

**Lyzozyme M1**	**WRi 012650**	**WORiA**	**F - GACTTTATGGCAGTATACCGA**
**(Lyz)**			**R - TGTTCCGTTGAATTTGTTCC**

**DNA**	**WRi 005640**	**WORiB**	**F - CTTAAATGACCATCAACCACAG**
**Methyltransferase (MTase)**			**R - GCTTCAATCAGGGAATTTGG**

**Contractile Tail**	**WRi 006970**	**WORiC**	**F- GTTGATGGTAGAGGTTATGCAG**
**Tube Protein**			**R - GAATATCCATACCACCAGCTC**

Reactions were performed in low profile 48-well white plates with flat cap strips (Bio-Rad). Ten microliter reactions included 400nM of each forward and reverse primer, 5 μl of 2× Dynamite qPCR mastermix (Molecular Biology Service Unit - University of Alberta) which included SYBR green (Molecular Probes) and Platinum Taq (Invitrogen), and 125ng of DNA. The thermal cycling conditions were 95°C for 2 minutes, 40 cycles of 95°C, 55°C, and 72°C for 30 seconds each, and a final 2 minute 72°C extension. Fluorescent data were acquired after every 72°C extension. A 60-95°C melting curve was performed to confirm the specificity of the products. No template controls were included to account for DNA contamination. All samples were analyzed in technical and biological triplicates. Standard curves were constructed through a dilution series to validate the primer pairs; all primers were found to have efficiencies that were roughly equal, using the equation E = 10^(-1/slope) ^-1, and suitable for relative comparisons, and r^2 ^values >0.99. The primer sequences were designed using PerlPrimer v1.1.14 [http://perlprimer.sourceforge.net] and are described in table 1. All primers were synthesized by Integrated DNA Technologies and were purified by standard desalting. PCR products were sequenced to confirm specificity of the primers and all amplified a single, specific target. Data were analyzed by the Opticon Monitor 3 software (Bio-Rad) which uses the ΔCT method. The average copy number of integrated phage was compared to the expected number based on published sequence data and the difference was statistically analyzed with a two-tailed t-test. The correlation tests between the three WO phages and *wRi *were performed using the Pearson Product Moment Correlation test. When determining the relative copy number for each of the phage types, it was assumed that integrated prophage sequences would amplify with the same efficiency as sequences from mature virus particles.

### Sequence analysis

Annotated genomes of *Wolbachia *strains *w*Mel [GenBank:NC_002978] [10] and *w*Ri [GenBank:NC_012416] [4], and phage strains WOCauB2 [GenBank:AB478515] [9], and WOVitA [GenBank:HQ906662] [12] were retrieved [22]. The phage regions [WRi_005250-005970] (WORiB) and [WRi_006570-WRi_007250] (WORiC) from the *w*Ri genome were used for whole phage genome alignments. The region [WD0562-WD0646] from the *w*Mel genome was used for WOMelB genome alignments. Whole genome comparisons were performed using the Mauve plug-in v.2.2.0 [20] for Geneious v5.4.4 [23]. The predicted amino acid sequences for the large terminase subunit and baseplate assembly gene W were used for phylogenetic analysis.

Proteins were aligned using the ClustalW multiple alignment algorithm implemented in Geneious v5.4.4. [23]. Model selection was performed using Prottest 2.4 [24] with Akaike's information criterion (AIC) used to select for an appropriate evolutionary model for each data set [terminase (JTT+I+Γ+F) and baseplate assembly protein W (JTT+Γ)] prior to analysis. The evolutionary history was inferred for both genes using the maximum likelihood method. Phylogenetic trees generated by PHYML used 1000 bootstrap replicated datasets and estimated gamma distribution and proportion of invariable sites [25].

## Results

### Presence and activity of WO prophages in *Wolbachia *of *D. simulans*

When lytic viruses replicate and lyse host cells, they do so through an enzymatic process involving a two component cell lysis system of a holin and lysozyme [26]. To date, there is no direct evidence that the WO phages of *w*Ri are capable of enzymatic lysis of bacterial hosts. Therefore, the term "lytic" is not used here to describe phage or phage DNA detected in excess of the integrated prophage genomes. Instead, replicating WO is referred to as a mature, extrachromosomal, or active phage. WO phages in *w*Mel and *w*Ri have been classically referred to as WO-A, WO-B, and WO-C [4,10]. However, the less ambiguous nomenclature WORiA, WORiB, and WORiC respectively is used here to describe the prophage types in *w*Ri and WOMelA and WOMelB for prophage types in *w*Mel.

Quantitative PCR was used to test whether *Wolbachia *prophages were replicating extrachromosomally. Specific primers that differentiate between the prophage types in *w*Ri were designed (table 1) and *Wolbachia *titer was determined by comparing the *wsp *gene copy number to the *Drosophila *nuclear *sod *gene. Integrated and extrachromosomal viral copy numbers were determined using primers specific to *Wolbachia *genes *lysozyme *(WORiA), *MTase *(WORiB), and *tail tube protein *(WORiC). The amplification of the WO-specific primers was compared to *Wolbachia *copy number using *wsp *(*w*Ri-specific primers).Values reported are the combination of integrated plus extrachromosomal phages.

**Figure 1:**
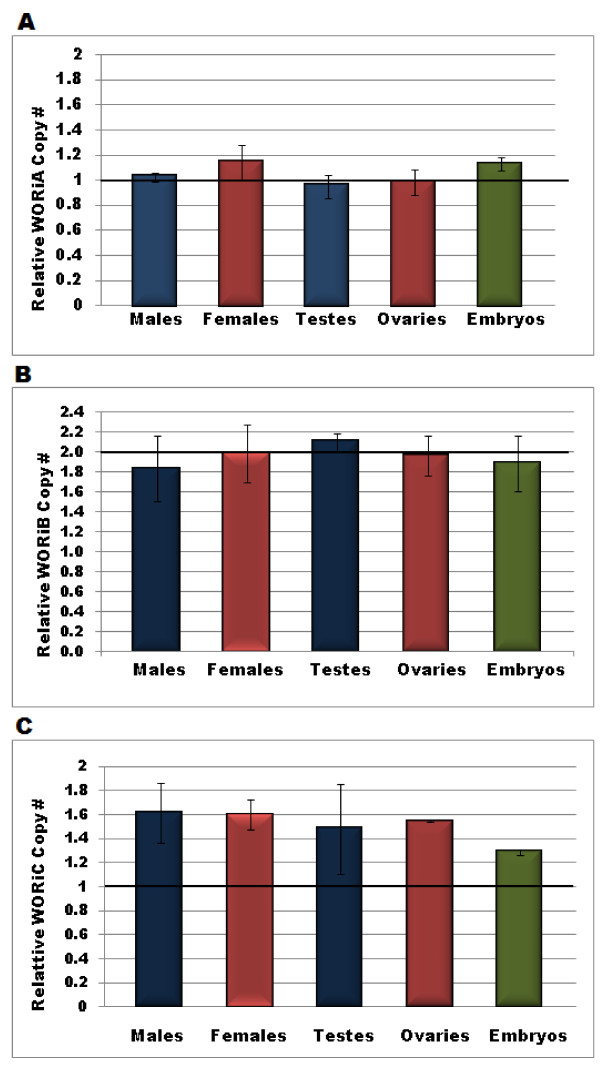
**Relative copy number of WO in males, females, testes, ovaries, and early embryos**. Relative copy number of ORFs encoding genes for lysozyme, MTase, and tail tube protein were measured by qPCR to determine the amount of extrachromosomal WORiA, WORiB, and WORiC, respectively in males, females, testes, ovaries, and embryos. The black line depicts the expected copy number for each of the phage types; one for A and C, and two for B. Of the three phage types, only WORiC is present in any extrachromosomal copies (p < 0.05). Error bars represent one standard deviation.

WORiA is found once in the *w*Ri genome. The relative copy number of the ORF which encodes a putative *lyzozyme *[WRi _012650] was measured in young males and females (three replicates of 15 flies each), testes and ovaries, and 15 minute AEL embryos. The relative lyzozyme (WORiA) copy number in these tissues ranged from 0.94 - 1.16 per *Wolbachia *cell (figure 1A). This is consistent with the single integrated copy in the genome and indicates no extrachromosomal WORiA (all p values > 0.05; two-tailed t-test).

In *w*Ri, there are two integrated copies of the WORiB prophage and each contains one copy of the *MTase *gene [WRi_005640; WRi_010300] [4]. In DSR males, females, testes, ovaries, and two-hour embryos, the relative *MTase *copy number ranged from 1.83-2.10 and was not significantly different than two per *Wolbachia *genome (all p values > 0.05, two-tailed t-test) (figure 1B). There is no evidence of extrachromosomal WORiB phage genomes.

The gene encoding the phage tail tube protein is present once in the *w*Ri genome on the WORiC insert. In males, females, testes, ovaries, and 15 minute AEL embryos, the relative tail tube protein copy number was significantly greater than the expected one copy per *Wolbachia *genome (p < 0.05 in all cases, two- tailed t-test) (figure 1C). Therefore, WORiC is the extrachromosomal phage in *w*Ri. The average density of all samples tested ranged from 1.29 - 1.61 copies of WORiC per *wsp *copy.

**Figure 2:**
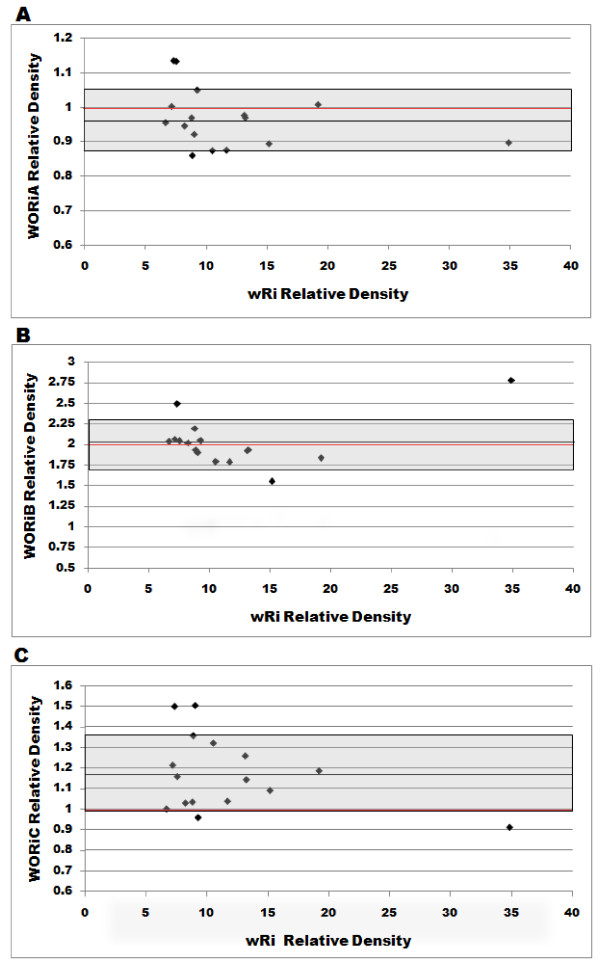
**Relative copy number of WO in 1hour synchronized 3' larvae individuals**. The relative copy numbers of each phage type are plotted against the relative density of *Wolbachia *in individual one hour synchronized third instar larvae. Each point on the graph represents one larva and the same 16 larvae were used to measure each of WORiA, WORiB, and WORiC. The shaded area represents one standard deviation of the combined 16 WO densities (0.085, 0.286, and 0.181, respectively) and the red line indicates the expected integrated copy number based on the published wRi genome sequence. The relative densities of *w*Ri and each of the WO phages did not show any significant correlation (Pearson; p > 0.05)

Occasionally, a DNA sample showed no evidence of extra-chromosomal WORiC DNA (data not shown). This indicates that DNA extracted from groups of flies may mask variation with respect to the amount of replicating phage per individual. Thus, third instar larvae were synchronized to a 1 hour age difference and *w*Ri, WORiA, WORiB, and WORiC numbers were measured for each individual to determine whether the WO copy number varied between individuals (figure 2). Relative phage densities were also compared to *Wolbachia *densities to determine whether variations in phage copy numbers were related to the bacterial density as observed by Bordenstein *et al *[15] in *N. vitripennis*. Among 16 third instar larvae tested, the *Wolbachia *densities ranged from 6.67 to 19.21 copies per host *sod *gene, with the exception of one outlier at 34.88. WORiA relative numbers averaged 0.97 and varied from 0.86 to 1.13 copies per *Wolbachia*. WORiB densities for the larvae averaged 2.02 copies per *w*Ri and ranged between 1.56 and 2.78. Finally, WORiC copy numbers averaged 1.17 and ranged between 0.91 and 1.50 per *wsp*. None of the densities of the three phage types correlated significantly with the *Wolbachia *density (Pearson correlation; p = 0.256, 0.12, and 0.16 for WORiA, WORiB, and WORiC, respectively) among the 16 samples tested. Removing the outlier individual (34.88 *Wolbachia *per host cell) from the analyses did not change the statistical outcome of the correlation test in WORiC (Pearson correlation; p > 0.7).

### Comparative genomics and phylogenetic analysis

The genome of the WORiC prophage is predicted to be 77,261 bp containing 56 ORFs [WRi _006570 to WRi_007250]. The core genome containing a DNA packaging and head assembly module and a tail morphogenesis module is shown in table 2 and is 24.2 kbp [WRi_006910 to WRi_007210]. The 35% GC content is identical to the GC content of the *w*Ri genome indicating a long period of co-evolution between prophage and bacteria.

**Table 2:** the conserved core tail morphogenesis and DNA packaging and head assembly regions of WORiC

Locus Tag	Open Reading Frame	Region
**WRi 006910**	**tail protein D, putative CDS**	
	
**WRi 006920**	**tail protein X, putative CDS**	
	
**WRi 006930**	**tail protein U, putative CDS**	
	
**WRi 006940**	**tail tape measure protein CDS**	
	
**WRi 006950**	**hypothetical protein CDS**	
	
**WRi 006960**	**hypothetical protein CDS**	
	
**WRi 006970**	**contractile tail tube protein CDS**	
	
**WRi 006930**	**tail protein U, putative CDS**	
	
**WRi 006940**	**tail tape measure protein CDS**	
	
**WRi 006950**	**hypothetical protein CDS**	
	
**WRi 006960**	**hypothetical protein CDS**	
	
**WRi 006970**	**contractile tail tube protein CDS**	
	
**WRi 006980**	**phage tail sheath protein CDS**	**Tail Morphogenesis**
	
**WRi 006990**	**hypothetical protein CDS**	
	
**WRi 007000**	**hypothetical protein CDS**	
	
**WRi 007010**	**hypothetical protein CDS**	
	
**WRi 007020**	**VrlC.2 CDS**	
	
**WRi 07030(a)**	**VrlC.1 CDS**	
	
**WRi 007040**	**transposase, IS5 family CDS**	
	
**WRi 07030(b)**	**VrlC.1 CDS**	
	
**WRi 007060**	**hypothetical protein CDS**	
	
**WRi 007070**	**Tail protein I, putative CDS**	
	
**WRi 007080**	**baseplate assembly protein J, putative CDS**	
	
**WRi 007090**	**baseplate assembly protein W, putative CDS**	
	
**WRi 007100**	**hypothetical protein CDS**	
	
**WRi 007110**	**baseplate assembly protein V CDS**	
	
**WRi 007120**	**hypothetical protein CDS**	

**WRi 007130**	**minor tail protein Z, putative CDS**	
	
**WRi 007140**	**hypothetical protein CDS**	
	
**WRi 007150**	**hypothetical protein CDS**	
	
**WRi 007160**	**hypothetical protein CDS**	
	
**WRi 007170**	**minor capsid protein C, putative CDS**	**DNA packaging and head assembly**
	
**WRi 007180**	**portal protein, lambda family CDS**	
	
**WRi 007190**	**phage uncharacterized protein CDS**	
	
**WRi 007200**	**hypothetical protein CDS**	
	
**WRi 007210**	**terminase large subunit, putative CDS**	

**Figure 3:**
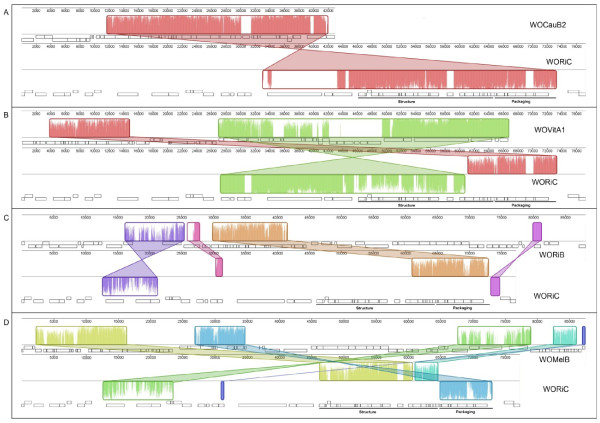
**Whole genome comparisons between WORiC, WOCauB2, WOVitA1, WOMelB, and WORiB**. Genomes of WORiC to A) WOCauB2 B) WOVitA1 C) WOMelB and D) WORiB are compared. Degree of sequence similarity is represented by the color intensity within each block. Areas of white within blocks indicate dissimilarity including gene insertions or deletions (see text). Each colored block represents a conserved region of homologous sequence between genomes. The placement of a block below the center axis indicates inverted regions.

The only confirmed WO mature virus particles that have been sequenced belong to *Wolbachia *of *Cadra cautella*, WOCauB2 and WOCauB3 [9,12]. More recently, Kent *et al *[12] used microarrays to capture the sequences of WOVitA and WOVitB which are the active phages in *w*VitA and *w*VitB respectively, infecting *N. vitripennis*. In this study, genomes from active phages were compared to WORi phage genomes to determine whether conserved regions are present in all active phages. Figure 3 shows the overall gene synteny between the WO phages. The heights of the colored peaks represent the degree of nucleotide similarity between collinear genomes. Pairwise alignments were performed between WORiC and WOCauB2 (figure 3a), WORiC and WOVitA1 (figure 3b), WORiC and WORiB (figure 3c) and WOMelB (figure 3d). Detailed lists of ORF alignments are included in the Additional file 1, Table S1, Additional file 2, Table S2, Additional file 3, Table S3, Additional file 4, Table S4, respectively. The WOMelB sequence used for comparisons included the upstream adjacent pyocin region identified by Wu *et al *[10]. These comparisons revealed conserved regions of homologous sequence and identified rearrangements and inversions between the genomes. The genes encoding putative structural and packaging proteins are present in two adjacent and conserved regions in WORiC, WOVitA1 and WOCauB2. WORiA and WOMelA did not align with other WO phage genomes (data not shown).

Comparisons between WORiC and WOCauB2 reveal a single block of homologous sequences spanning the structural and packaging regions (figure 3a). There are three separate areas of dissimilarity between WORiC and WOCauB2. These include two transposable elements and an uncharacterized phage protein [WRi_007190].

Notable areas of dissimilarity between WOVitA1 and WORiC (white areas; figure 3b) include two transposable elements [WRi_006820] interrupting an ankyrin repeat protein gene [WRi_006810, WRi_p06840]. Genome alignments were also used to assign possible functions to previously annotated hypothetical ORFs. A hypothetical gene, [WRi_p07030], shares 74.7% pairwise identity to the virulence protein gene VrlC.1 of WOVitA1 and is pseudonized by the transposon insertion [WRi_007040]. The annotated hypothetical protein [WRi _007070] is homologous to tail protein I from WOVitA1 (96%, 3e-143). The major region of dissimilarity between WOVitA1 and WORiC could be a result of horizontal gene transfer into WOVitA1 or gene loss in WORiC. These ORFs in WOVitA1 encode MutL and three transcriptional regulators [ADW80184.1, ADW80182.1 to ADW80179.1]. Although WOVitA1 and WORiC share 36 homologs compared to 33 shared between WORiC and WOCauB2, WORiC is more similar to WOCauB2 (92.4%).

The WORiB genome shares only the ORFs found within the packaging region [WRi_005460 to WRi_005610] with WORiC (figure 3c). However, when the pyocin sequences, containing the viral structural genes, are included in the WOMelB genome and aligned with WORiC, the structural and packaging regions are conserved, but rearranged in WOMelB compared to WORiC (figure 3d).

**Figure 4:**
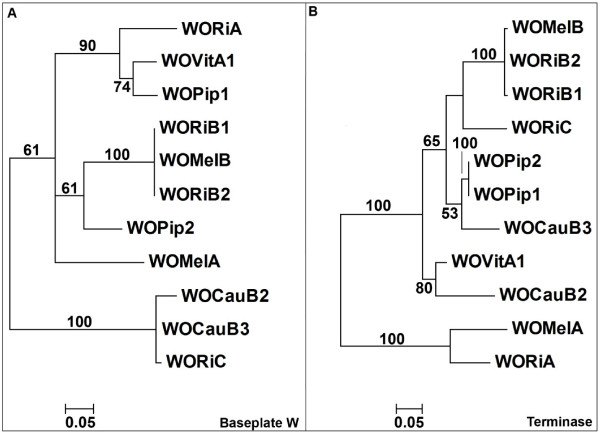
**Phylogeny of terminase and baseplate assembly protein W amino acid sequences**. Maximum-likelihood phylogeny based on translated amino-acid sequences of A) baseplate assembly gene W (tail morphogenesis module) and B) large terminase subunit gene (DNA packaging and head assembly module) of *Wolbachia *WO phages from published genomes. Bootstrap values for each node are based on 1000 resamplings.

The evolutionary relationships of the tail morphogenesis module and head assembly and DNA packaging module were examined by phylogenetic analysis. Phylogenetic trees based on baseplate assembly protein W and the large terminase subunit showed different evolutionary relationships for related phages, with the exception of the WOMelB, WORiB1 and WORiB2 clade (figure 4). WORiC shows the greatest phylogenetic relatedness to WOCauB2 and WOCauB3 for baseplate assembly protein W (figure 4a), which is reflected by the degree of nucleotide similarity in the alignment (figure 3a). In contrast, the large terminase subunit of WORiC is most closely related to the *w*Mel and *w*Ri B-type phages (figure 4b).

## Discussion

### WORiC is the active phage in *w*Ri

When temperate bacteriophages infect sensitive bacteria their viral genomes direct DNA replication of the phage, cell lysis and the release of progeny, or, if the lytic state is suppressed, they integrate into the bacterial chromosome in the form of a prophage, in what is known as the lysogenic state. In *Escherichia coli*, lambdoid prophages are stably integrated into the host chromosome and do not undergo lytic induction until the bacterial SOS response is activated [27]. Gavotte *et al *[17] used a filtration-based purification method accompanied by TEM and ORF7-specific PCR to show that mature phage particles form in *Wolbachia*-infected tissues in both *D. simulans *and *D. melanogaster*, but the specific identity of these virus particles and the regulation of their induction was not addressed.

In this study, the activity of the three distinct prophages found in *w*Ri infecting *D. simulans *was measured using quantitative PCR. Phage type-specific primers were used to determine how many copies of the phage genomes were present in addition to the integrated forms. The only phage chromosome to appear in excess of the integrated copy number was WORiC. The average number of copies of WORiC in all tissues tested ranged from 1.29 - 1.61 copies per *Wolbachia*, consistently above the one copy integrated into the *w*Ri genome. Thus, WORiC appears to be the only actively replicating phage in *D. simulans*.

*w*Ri is considered to be a high CI strain of *Wolbachia *in *D. simulans*; embryonic lethality resulting from crosses between infected males and uninfected females is typically between 90 - 100% [28,29]. In *N. vitripennis *infected with *w*VitB, which is also a high CI-inducing strain of *Wolbachia*, Bordenstein *et al *[15] reported an average WOVitB copy number of 1.6 ± 0.12 per *Wolbachia*. In the present study, a similar relative density of WORiC suggests that this phage is the active virus observed in past TEM micrographs of *Drosophila *tissues [5,17]. WORiC genes have been reported as actively transcribed in previous literature. Specifically, the ankyrin related genes in WORiC are expressed in males, females, ovaries, testes, early (2 hour AEL) and late (overnight) embryos [4].

### WORiB and WORiA are non-functional phage remnants

WORiA and WORiB did not show any evidence of extrachromosomal DNA beyond the one and two copies, respectively, found within the *w*Ri genome. Alignments to WOCauB and WOVitA1 show that both WORiA and WORiB lack the core structural components necessary for virion assembly. The persistence of WORiA and WORiB within the *w*Ri genome suggests that there may be selective pressures maintaining these two prophages. There is evidence that WORiB is actively transcribing at least one ORF located within the prophage genome [30] and so this region may be necessary for another, unrelated, aspect of *Wolbachia *biology.

### Phage density remains consistent among time-synchronized cohorts and does not correlate with *Wolbachia *density in larvae

The average density of WORiC was derived from pooled samples of multiple individuals and tissues. When 16 third instar larvae were individually measured for phage density, WORiA and WORiB did not significantly deviate from the expected means of one and two copies, respectively. Individual larva, however, had a much wider distribution of WORiC copy numbers, ranging from individuals that appeared to have no extrachromosomal viruses to individuals having more than 1.5 WORiC per *Wolbachia*. This indicates that not every individual within the larval population is experiencing viral replication, although most are. Currently, the signals which induce viral replication within the confines of an endosymbiotic bacterium are unknown.

Along with the WO density in individual third instar larvae, the relative *Wolbachia w*Ri density per *D. simulans *host cell was also measured. The *w*Ri density did not significantly correlate with WORiA, WORiB, or WORiC relative densities. However, the WORiC density trends toward a slight inverse association with *w*Ri density. It is possible that with a larger sample population, more statistical significance would emerge. This lack of correlation does not refute the phage density model postulated by Bordenstein *et al *[15], whereby the *Wolbachia *copy number and CI in *N. vitripennis *was found to be inversely related to phage activity. Rather, it raises the notion that phage density is a population and strain-specific factor. Low levels of replicating phage, as seen here for WORiC, may not significantly impact *Wolbachia w*Ri density and the strength of CI in *Drosophila*. The effect of phage copy number on CI level in *D. simulans *has yet to be examined.

### Comparative Genomics and phylogenetics of *Wolbachia *bacteriophages

Since WORiC in this study was the only *w*Ri prophage capable of extrachromosomal replication, a comparative genomic approach was taken to identify the core genome conserved between WORiC and two known temperate bacteriophages WOVitA1 and WOCauB2. This approach identified essential regions required for phage generation. The genomes of WORiC, WOVitA1, and WOCauB2 show considerable sequence homology which supports the view that WORiC is the active form of phage in *w*Ri. In contrast, the WORiB genome and the WOMelB genome lacking the upstream pyocin region share few homologous sequences with WORiC. Genes with sequence homology in WORiB, WOMelB, and WORiC belong to the DNA packaging and head assembly region. However, the core structural/tail region of WORiC aligns with WOMelB once the pyocin region is included in the analysis. WORiB lacks the pyocin-like region and is therefore deficient in most tail morphogenesis genes.

The chimeric nature of WO phages was initially described by Masui *et al *[6], who identified the large terminase subunit, portal protein and minor capsid protein of the packaging region in WOKue as lambda-like, and the baseplate assembly proteins of the structural region as P2-like. This hybridization of lambda and P2 sequences is not exclusive to WO phages, since chimeric phages have been described in other systems; for example *Xylella fastidiosa *phages XfP1 and XfP2 are also lambda/P2 chimeras [16]. Due to recombination and genetic mosaicism, different parts of a bacteriophage genome can have different evolutionary histories [31]. In the chimeric WO phages (figure 4), the large terminase subunit sequence from the DNA packaging and head assembly regions shows a different phylogenetic relationship than the baseplate assembly protein W sequence from the tail morphogenesis regions. This modular nature of WO phages has been described previously [19].

The two conserved modules shared by WORiC and the temperate phages WOCauB2 and WOVitA1 include the DNA packaging and head assembly region and the tail morphogenesis region. The genome encoding the DNA packaging and head assembly module includes ORFs that putatively code for a portal protein, a minor capsid protein and the large subunit of the terminase protein. This large terminase subunit contains a DNA-dependent ATPase domain and site-specific nuclease domain which are both involved in DNA translocation during packaging. In double stranded DNA phages, terminases are generally accompanied by a small subunit involved in DNA binding [32,33]. However, no homolog of this small subunit has been identified in any WO genome. The portal protein of tailed bacteriophages forms a complex with the terminase proteins which translocates phage DNA into the prohead during phage replication [33]. The conservation of these packaging genes suggests that DNA packaging in WO phages is driven by an ATP-dependent DNA translocation motor similar to other tailed bacteriophages.

Similarly, the organization of the tail morphogenesis module is conserved among WOVitA, WOCauB, and WORiC. Genes involved in tail assembly include the tail proteins, tail tape measure protein, the tail sheath protein, the contractile tail tube protein and baseplate assembly proteins J,W, and V. Tail morphogenesis in the subfamily Myoviridae, which have long contractile tails, is the most complex of all tailed bacteriophages. In the Myoviridae T4, P2 or Mu, baseplate assembly occurs first and is required for sheath and tail polymerization. It is from the baseplate that the tube polymerizes to a length determined by the tail-tape measure protein and this is followed by the tail sheath which extends the length of the tail [34].

The presence of the tail sheath gene in active WO genomes suggests that, with respect to tail structure and assembly, these phages are more similar to Myoviridae than to the subfamily Siphoviridae, which includes lambda and lacks contractile tails. The phage tail mediates genome delivery into host cells, and is required for the generation of infectious phages. The absence of this region in the WORiB genome may contribute to the inability of WORiB to form infectious particles.

Unlike WORiC, where the packaging region is located adjacent to the structural proteins, in WOMelB the structural proteins are divided in the genome and separated from the packaging region by approximately 18kbp [35]. One region of structural genes found in WOMelB was initially characterized as a pyocin-like region. Therefore, active phage generation in *D. melanogaster w*Mel could result from the coordinate replication of both packaging and structural regions. Despite much previous interest in *Wolbachia*'s ankyrin containing genes [35,36], and the suggestion that they may influence phage function, the ORFs encoding ankyrin-containing motifs are outside the core conserved regions of WORiC, WOVitA1 and WOCauB3. The role of ankyrin coding genes in the WO-*Wolbachia*-host relationship remains elusive [37,38].

Our results suggest that *Wolbachia *phages WORiC and known active phages WOCauB and WOVitA1 represent a conserved class of *Wolbachia *phages. Interest in the conserved genetic modules of the lambda-like DNA packaging and head assembly genes and P2-like tail morphogenesis genes led to the investigation of the relatedness of the *Wolbachia *phages. Phylogenetic analysis shows similarity between WORiC and WO-B's found in *w*Mel and *w*Ri (based on large terminase subunit phylogeny) and similarity between WORiC and WOCauB2 and WOCauB3 (based on the baseplate assembly protein W phylogeny). These divergent topologies are indicative of the horizontal transfer events occurring between phage genomes. Similarity of genomes of active WO phages may be due to the fact that they have a common, recent origin, or because active WO phages are operating within a limited framework of endosymbiotic bacteria, where opportunities for incorporating novel gene sequences by recombination are limited. Given the present level of knowledge of active WO bacteriophages, we cannot distinguish between these and other possible evolutionary scenarios.

## Conclusions

The genome of WORiC shares two main regions of similarity to WO phages infecting *w*Cau and *w*Vit. These two regions encode DNA packaging and head assembly proteins and tail morphogenesis and structural proteins. The conserved structural and packaging regions appear to be necessary for generation of mature virus particles; all active WO phages characterized to date contain these homologous components.

The obligate intracellular nature of *Wolbachia *makes detailed examination of WO and its temperate lifestyle a challenge. Here, a phage-specific quantitative PCR approach was employed to determine that WORiC is the active prophage element in *w*Ri. On an organismal and tissue-specific level, WORiC is present in very low densities; this low density is expected in *w*Ri's high CI environment and is consistent with the phage density model developed in *Nasonia *[15]. On an individual basis, however, no correlation was found between *w*Ri and WO phage density in synchronized third instar larvae. This study provides an integrated computational and molecular approach to investigate the complex biology of the host insect, *Wolbachia *endosymbiont, and WO bacteriophage.

## Authors' contributions

JAB and CLG performed the qPCR experiments, PDB carried out the alignments and *in silico *analysis, JAB, PDB, and HLH conceived all experiments and analyzed the data and all authors wrote the manuscript. All authors have read and approved the final manuscript.

## Supplementary Material

Additional file 1 **ORFs included in the whole genome alignment of WORiC and WOCauB2**. Highlighted regions match colours indicated in Figure 3a and represent regions of sequence similarity. Click here for file

Additional file 2 **ORFs included in the whole genome alignment of WORiC and WOVitA1**. Highlighted regions match colours indicated in Figure 3b and represent regions of sequence similarity. Click here for file

Additional file 3 **ORFs included in the whole genome alignment of WORiC and WORiB**. Highlighted regions match colours indicated in Figure 3c and represent regions of sequence similarity. Click here for file

Additional file 4 **ORFs included in the whole genome alignment of WORiC and WOMelB**. Highlighted regions match colours indicated in Figure 3d and represent regions of sequence similarity. Click here for file

## References

[B1] Lo N Casiraghi M Salati E Bazzochi C Bandi C How many *Wolbachia *supergroups exist? Molecular Biology and Evolution 2002 19 341 346 11861893 10.1093/oxfordjournals.molbev.a004087

[B2] Werren JH Zhang W Guo LR Evolution and phylogeny of *Wolbachia*: Reproductive parasites of arthropods Proceedings of the Royal Society B 1995 261 53 63 10.1098/rspb.1995.0117 7644549

[B3] Stouthamer R Breeuwer J Hurst G *Wolbachia pipientis*: microbial manipulator of arthropod reproduction Annual Review of Microbiology 1999 53 71 102 10.1146/annurev.micro.53.1.71 10547686

[B4] Klasson L Westberg J Sapountzis P Naslund K Lutnaes Y Darby AC Veneti Z Chen L Braig HR Garrett R The mosaic genome structure of the *Wolbachia w*Ri strain infecting *Drosophila simulans* Proceedings of the National Academy of Sciences USA 2009 106 14 5725 5730 10.1073/pnas.0810753106 PMC2659715 19307581

[B5] Masui S Sasaki T Ishikawa H Genes for the type IV secretion system in an intracellular symbiont, *Wolbachia*, a causative agent of various sexual alterations in arthropods Journal of Bacteriology 2000 182 22 6529 6531 10.1128/JB.182.22.6529-6531.2000 11053403 PMC94805

[B6] Masui S Kuroiwa H Sasaki T Inui M Kuroiwa T Ishikawa H Bacteriophage WO and virus-like particles in *Wolbachia*, an endosymbiont of arthropods Biochemical and Biophysical Research Communications 2001 283 5 1099 1104 10.1006/bbrc.2001.4906 11355885

[B7] Klasson L Walker T Sebaihia M Sanders MJ Quail MA Lord A Sanders S Earl J O'Neill SL Thomson N Genome evolution of *Wolbachia *strain *w*Pip from the *Culex pipiens *group Molecular Biology and Evolution 2008 25 9 1877 1887 10.1093/molbev/msn133 18550617 PMC2515876

[B8] Salzberg SL Puiu D Summer DD Nene V Lee NH Genome sequence of the *Wolbachia *endosymbiont of *Culex quinquefasciatus *JHB Journal of Bacteriology 2009 191 5 1725 10.1128/JB.01731-08 19114486 PMC2648186

[B9] Tanaka K Furukawa S Nikoh N Sasaki T Fukatsu T Complete WO phage sequences revealed their dynamic evolutionary trajectories and putative functional elements required for integration into *Wolbachia *genome Applied and Environmental Microbiology 2009 75 17 5676 5686 10.1128/AEM.01172-09 19592535 PMC2737910

[B10] Wu M Sun LV Vamatheven J Riegler M Deboy R Brownlie JC McGraw EA Martin W Esser C Ahmadinejad N Phylogenomics of the reproductive parasite *Wolbachia pipientis w*Mel: A streamlined genome overrun by mobile genetic elements PLoS Biology 2004 2 3 0327 10.1371/journal.pbio.0020069 PMC368164 15024419

[B11] Fujii Y Kubo T Ishikawa H Sasaki T Isolation and characterization of the bacteriophage WO from *Wolbachia*, an arthropod endosymbiont Biochemical and Biophysical Research Communications 2004 317 1183 1188 10.1016/j.bbrc.2004.03.164 15094394

[B12] Kent B Salichos L Gibbons J Rokas A Newton I Clark M Bordenstein SR Complete bacteriophage transfer in a bacterial endosymbiont (*Wolbachia*) determined by targeted genome capture Genome Biology and Evolution 2011 3 209 218 10.1093/gbe/evr007 21292630 PMC3068000

[B13] Bordenstein SR Wernegreen JJ Bacteriophage flux in endosymbionts (*Wolbachia)*: Infection frequency, lateral transfer and recombination rates Molecular Biology and Evolution 2004 21 10 1981 1991 10.1093/molbev/msh211 15254259

[B14] Ishmael N Dunning Hotopp JC Ioannidis P Biber S Sakomoto J Siozios S Nene V Werren J Bourtzis K Bordenstein SR Extensive genomic diversity of closely related *Wolbachia *strains Microbiology 2009 155 7 2211 2222 10.1099/mic.0.027581-0 19389774 PMC2888116

[B15] Bordenstein SR Marshall ML Fry AJ Kim U Wernegreen JJ The tripartite associations between bacteriophage, *Wolbachia*, and arthropods PLoS Pathogens 2006 2 5 e43 10.1371/journal.ppat.0020043 16710453 PMC1463016

[B16] Canchaya C Proux C Fournous G Bruttin A Brussow H Prophage Genomics Microbiology and Molecular Biology Reviews 2003 67 2 238 276 10.1128/MMBR.67.2.238-276.2003 12794192 PMC156470

[B17] Gavotte L Vavre F Henri H Ravallec M Stouthamer R Bouletreau M Diversity, distribution and specificity of WO phage infection in *Wolbachia *of four insect species Insect Molecular Biology 2004 13 2 147 153 10.1111/j.0962-1075.2004.00471.x 15056362

[B18] Sanogo YO Dobson SL WO bacteriophage transcription in *Wolbachia-*infected *Culex pipiens* Insect Biochemistry and Molecular Biology 2005 36 80 85 10.1016/j.ibmb.2005.11.001 16360953

[B19] Kent B Bordenstein SR Phage WO of *Wolbachia*: lambda of the endosymbiont world Trends in Microbiology 2010 18 4 173 181 10.1016/j.tim.2009.12.011 20083406 PMC2862486

[B20] Casjens S Prophages and bacterial genomics: what have we learned so far? Molecular Microbiology 2003 49 277 300 10.1046/j.1365-2958.2003.03580.x 12886937

[B21] Zhou WG Rousset F O'Neill SL Phylogeny and PCR-based classification of *Wolbachia *strains using *wsp *gene sequences Proceedings of the Royal Society B 1998 265 509 515 10.1098/rspb.1998.0324 9569669 PMC1688917

[B22] Benson DA Karsch-Mizrachi I Lipman DJ Ostell J Wheeler DL GenBank Nucleic Acids Research 2008 36 Database issue D25 30 18073190 10.1093/nar/gkm929 PMC2238942

[B23] Drummond A Ashton B Buxton S Cheung M Cooper A Duran C Field M Heled J Kearse M Markowitz S Geneious 5.4 2011 http://www.geneious.com

[B24] Abascal F Zardoya R Posada D ProtTest: Selection of best-fit models of protein evolution Bioinformatics 2005 21 2104 2105 10.1093/bioinformatics/bti263 15647292

[B25] Guindon S Gascuel O A simple, fast, and accurate algorithm to estimate large phylogenies by maximum likelihood Sytematic Biology 2003 52 696 704 10.1080/10635150390235520 14530136

[B26] Srividhya KV Krishnaswamy S Sub classification and targeted characterization of prophage-encoded two-component lysis cassette Journal of Biosciences 2007 32 5 979 990 17914239 10.1007/s12038-007-0097-x

[B27] Little J Michalowski C Stability and instability in the lysogenic state of phage Lambda Journal of Bacteriology 2010 192 23 6064 6076 20870769 10.1128/JB.00726-10 PMC2976446

[B28] Hoffmann AA Turelli M Simmons GM Unidirectional incompatibility between populations of *Drosophila simulans* Evolution 1986 40 692 701 10.2307/2408456 28556160

[B29] Veneti Z Clark ME Zabalou S Karr TL Savakis C Bourtzis K Cytoplasmic incompatibility and sperm cyst infection in different *Drosophila-Wolbachia *associations Genetics 2003 164 2 545 552 12807775 10.1093/genetics/164.2.545 PMC1462605

[B30] Saridaki A Sapountzis P Harris HL Batista P Biliske J Pavlikaki H Oehler S Savakis C Braig HR Bouchon D *Wolbachia *prophage DNA adenine methyltransferase genes in different *Drosophila*-*Wolbachia *associations PLoS One 2011 6 5 10.1371/journal.pone.0019708 PMC3089641 21573076

[B31] Lawrence JG Hatfull GF Hendrix RW Imbroglios of viral taxonomy: Genetic exchange and failings of phenetic approaches Journal of Bacteriology 2002 184 17 4891 4895 10.1128/JB.184.17.4891-4905.2002 12169615 PMC135278

[B32] Black L DNA packaging in dsDNA bacteriophages Annual Review of Microbiology 1989 43 267 292 10.1146/annurev.mi.43.100189.001411 2679356

[B33] Rao VB Feiss M The bacteriophage DNA packaging motor Annual Review of Genetics 2008 42 647 681 10.1146/annurev.genet.42.110807.091545 18687036

[B34] Leiman PG Arisaka F van Raaij MJ Kostyuchenko VA Aksyuk AA Kanamaru S Rossmann MG Morphogenesis of the T4 tail and tail fibers Virology Journal 2010 7 355 10.1186/1743-422X-7-355 21129200 PMC3004832

[B35] Iturbe-Ormaetxe I Burke GR Riegler M O'Neill SL Distribution, expression, and motif variability of ankyrin domain genes in *Wolbachia pipientis* Journal of Bacteriology 2005 187 15 5136 5145 10.1128/JB.187.15.5136-5145.2005 16030207 PMC1196006

[B36] Duron O Boureux A Echaubard P Berthomieu A Berticat C Fort P Weill M Variability and Expression of ankyrin domain genes in *Wolbachia *variants infecting the mosquito *Culex pipiens* Journal of Bacteriology 2007 180 12 4442 4448 10.1128/JB.00142-07 PMC1913362 17449622

[B37] Walker T Klasson L Sebaihia M Sanders MJ Thomson N Parkhill J Sinkins SP Ankyrin repeat domain-encoding genes in the wPip strain of *Wolbachia *from the *Culex pipiens *group BMC Biology 2007 5 39 10.1186/1741-7007-5-39 17883830 PMC2045654

[B38] Yamada R Iturbe-Ormaetxe I Brownlie JC O'Neill SL Functional test of the influence of *Wolbachia *genes on cytoplasmic incompatibility expression in *Drosophila melanogaster* Insect Molecular Biology 2010 20 1 75 85 20854481 10.1111/j.1365-2583.2010.01042.x

